# A yeast's eye view of mammalian reproduction: cross-species gene co-expression in meiotic prophase

**DOI:** 10.1186/1752-0509-4-125

**Published:** 2010-09-06

**Authors:** Yunfei Li, Ka-sum Lam, Nairanjana Dasgupta, Ping Ye

**Affiliations:** 1 School of Molecular Biosciences, Washington State University, PO Box 647520, Pullman, WA 99164, USA; 2 Center for Reproductive Biology, Washington State University, Pullman, WA 99164, USA; 3 Department of Statistics, Washington State University, Pullman, WA 99164, USA

## Abstract

**Background:**

Meiotic prophase is a critical stage in sexual reproduction. Aberrant chromosome recombination during this stage is a leading cause of human miscarriages and birth defects. However, due to the experimental intractability of mammalian gonads, only a very limited number of meiotic genes have been characterized. Here we aim to identify novel meiotic genes important in human reproduction through computational mining of cross-species and cross-sex time-series expression data from budding yeast, mouse postnatal testis, mouse embryonic ovary, and human fetal ovary.

**Results:**

Orthologous gene pairs were ranked by order statistics according to their co-expression profiles across species, allowing us to infer conserved meiotic genes despite obvious differences in cellular synchronicity and composition in organisms. We demonstrated that conserved co-expression networks could successfully recover known meiotic genes, including homologous recombination genes, chromatin cohesion genes, and genes regulating meiotic entry. We also showed that conserved co-expression pairs exhibit functional connections, as evidenced by the annotation similarity in Gene Ontology and overlap with physical interactions. More importantly, we predicted six new meiotic genes through their co-expression linkages with known meiotic genes, and subsequently used the genetically more amenable yeast system for experimental validation. The deletion mutants of all six genes showed sporulation defects, equivalent to a 100% validation rate.

**Conclusions:**

We identified evolutionarily conserved gene modules in meiotic prophase by integrating cross-species and cross-sex expression profiles from budding yeast, mouse, and human. Our co-expression linkage analyses confirmed known meiotic genes and identified several novel genes that might be critical players in meiosis in multiple species. These results demonstrate that our approach is highly efficient to discover evolutionarily conserved novel meiotic genes, and yeast can serve as a valuable model system for investigating mammalian meiotic prophase.

## Background

Meiosis is essential for sexual reproduction in eukaryotes. It is a conserved process in which diploid cells undergo one round of DNA replication followed by two rounds of chromosome segregation to produce haploid cells. Meiosis I separates homologous chromosomes, while meiosis II is similar to mitosis, involving separation of sister chromatids to form haploid cells. Meiosis I and II are, in turn, each divided into four stages: prophase, metaphase, anaphase, and telophase [1].

**Figure 1:**
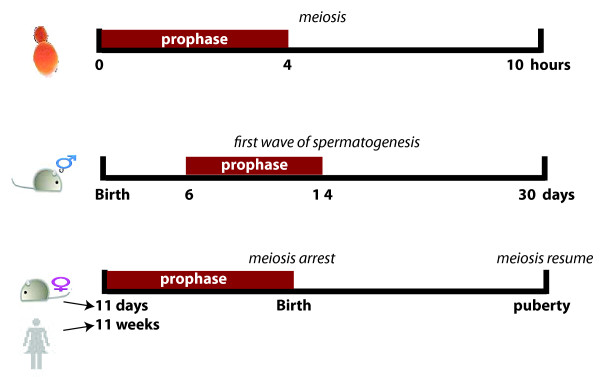
**Meiotic time frame varies by sex and species**. Yeast cells finish meiosis in 10 hours in a nutrient depleted sporulation medium. The first wave of spermatogenesis is complete within 30 days after birth. Female meiosis initiates during the embryonic stage, arrests before birth, and resumes after puberty. Meiotic prophase is labeled in the timetable of each species and sex.

General chromosome behavior during meiosis is conserved in a range of organisms from unicellular budding yeast to multi-cellular mammals [1,2]. However, the time frame required for each meiotic stage varies greatly by sex and species (Figure 1). Budding yeast with heterozygosity at the mating-type locus can finish meiosis in hours under a nutrient-depleted environment [3-5]. Whereas in mammals, germ cells in gonads may take from days to decades to accomplish meiosis with the support of neighboring somatic cells through hormonal cues [6-9]. Male meiosis occurs continuously and asynchronously from puberty. However, the first wave of spermatogenesis proceeds in a relatively synchronous fashion, which provides the perfect time to investigate genetic control in male meiosis [7,8]. In females, the entire oogonial population initiates meiosis synchronously in fetal ovaries and becomes arrested near the end of prophase I before birth. A small cohort of arrested oocytes then resumes meiosis during each ovulation after puberty [6,9-11]. Therefore, prophase I is the most synchronized stage of female meiosis.

The important function of meiosis is to employ recombination to generate genetic diversity in offspring. This process happens during meiotic prophase I (abbreviated as meiotic prophase in the rest of the paper). Each pair of homologous chromosomes aligns together, allowing genetic recombination to occur between non-sister chromatids. The exchanged DNA fragments result in new genetic combinations within chromosomes that will be passed to haploid cells [2]. Recombination errors cause mis-segregation of chromosomes and the production of aneuploid gametes, which are associated with human birth defects and miscarriages. Indeed, alterations in maternal meiotic recombination are an important contributor to both autosomal and sex chromosome trisomies in humans [12]. Many genes involved in meiotic prophase have been characterized in yeast [1]. But comparatively few mammalian genes are known to be involved in this process, and are mainly identified through gene targeting in mice and chromosomal analysis in patients with fertility disorders. Preliminary studies suggest that humans follow the yeast paradigm, with the early appearance of recombination and cohesion proteins during prophase [13,14].

The recent availability of reproductive tissue-specific expression profiles for humans and mice allows us to monitor gene co-expression and predict plausible new meiotic genes that are important in human reproduction [6-9,15,16]. However, the analyses have been mainly limited to simple clustering of expression profiles, which could pinpoint many meiotic gene candidates. Moreover, *in vivo *mammalian genetics is time-consuming to validate candidate meiotic genes. Here we propose to use budding yeast as a model system with which to identify conserved meiotic genes by applying an order statistics ranking method. Previous computational efforts have demonstrated the feasibility of identifying evolutionarily conserved functional modules through the mining of gene co-expressions, protein interactions, or phenotypes across species [17-19]. Stuart *et al *described the use of gene co-expression and metagenes to identify conserved genetic modules in humans, flies, worms, and yeast [19]. In this study, we conducted cross-species and cross-sex inferences to identify conserved co-expressed genes in meiotic prophase from time-series expression profiles in yeast, mice, and humans. We identified known meiotic genes from co-expression networks and predicted candidate meiotic genes from co-expression linkages with known meiotic genes. Several novel meiotic genes were subsequently validated in the tractable yeast system. We also examined conserved co-expression pairs using enriched genomic information in yeast. Our approach yielded novel genes during the critical meiotic prophase of sexual reproduction and provided insights into the molecular events leading to human reproductive defects.

## Results

### Construction of conserved gene co-expression networks for meiotic prophase

**Figure 2:**
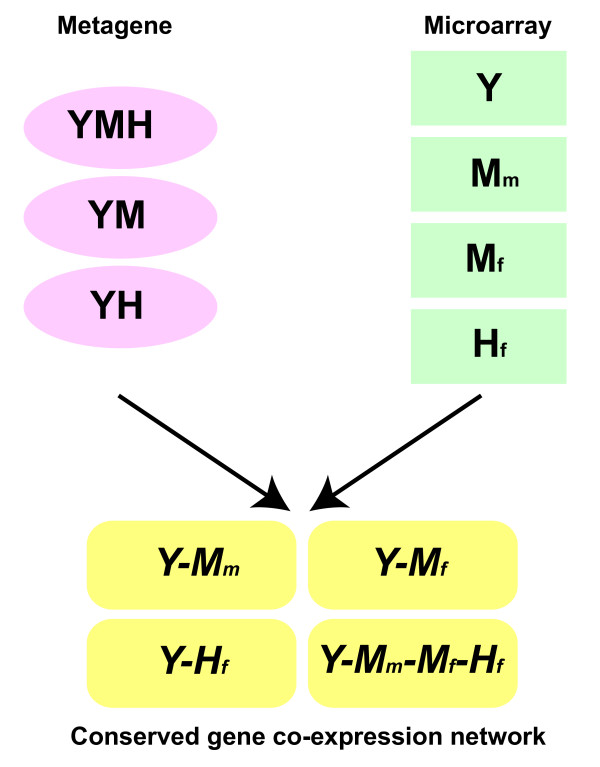
**The framework to derive conserved co-expression networks for meiotic prophase**. Three types of metagenes were compiled: YMH represents genes conserved among yeast, mice, and humans; YM represents genes conserved between yeast and mice; YH represents genes conserved between yeast and humans. Time-series microarray profiles for meiotic prophase are available from yeast (Y), mouse postnatal testis (M_m_), mouse embryonic ovary (M_f_), and human fetal ovary (H_f_). Gene co-expression networks were constructed by including metagene pairs showing co-expression across species. The network *Y-M_m _*was constructed using metagenes YMH and YM, and microarrays Y and M_m_. The network *Y-M_f _*was constructed using metagenes YMH and YM, and microarrays Y and M_f_. The network *Y-H_f _*was constructed using metagenes YMH and YH, and microarrays Y and H_f_. The network *Y-M_m_-M_f_-H_f _*was constructed using metagenes YMH and all four microarray studies.

There are at least two major challenges to studying meiotic prophase in mammals: the limited amount of available neonatal testis and fetal ovarian tissue and the limited number of techniques for manipulating germ cells *in vitro*. Microarray is one of the few high-throughput approaches that can provide valuable resources for probing meiotic pathways and networks. The critical issue is germ cell synchrony. Here, we focus on meiotic prophase in the first wave of spermatogenesis and in embryonic oogenesis, which is the most synchronized meiotic process. To construct a conserved co-expression network for meiotic prophase, we analyzed four time-series microarray studies in yeast (Y), mouse postnatal testis (M_m_), mouse embryonic ovary (M_f_), and human fetal ovary (H_f_) (Figure 2) [5,6,8,9].

We define metagenes as genes conserved across multiple species [19]. We derived three types of metagenes: metagenes conserved across yeast, mice, and humans (YMH), and metagenes conserved only between two species (YM and YH) (Table 1, Figure 2). Every metagene type contains yeast genes. Thus, we can examine conserved co-expression genes using the enormous amount of genomic data available on yeast and validate predicted genes using yeast as a tractable experimental system.

**Table 1:** The number of conserved genes in yeast, mouse, and human

	Metagene type*					
				
	YMH	YM	YH	*Sum*	*Genome coverage^#^*	*Genes per metagene*
Metagene	2,036	146	129	2,311	-	-
Yeast	2,124	151	131	2,406	42%	1.04
Mouse	2,121	151	-	2,272	10%	1.04
Human	2,165	-	133	2,298	10%	1.06

* YMH, YM, and YH are mutually exclusive metagene types.

^# ^The genome coverage was calculated based on total protein numbers in yeast (5,792), mouse (23,132), and human (22,983) [52].

The use of metagenes allows us to connect gene expression profiles across species. To identify conserved co-expression genes, we first computed the Pearson correlation of gene expression across the prophase time points for metagene pairs in each species. Metagene pairs were subsequently ranked according to Pearson correlation coefficients. A rank ratio was obtained for each metagene pair by dividing its rank by the total number of metagene pairs in the species. Next, we used the joint cumulative distribution of order statistics to evaluate the probability of observing a particular configuration of ranks across different organisms by chance [19]. This P-value quantifies the significance of observing the co-expression of metagene pairs across species. Metagene pairs with P-values greater than a threshold can be connected to form networks. In this way, we constructed four networks that capture gene co-expression during meiotic prophase conserved in different organisms and different sexes: *Y-M_m_*, *Y-M_f_*, *Y-H_f_*, and *Y-M_m_-M_f_-H_f _*(Figure 2). A toy example for constructing gene co-expression networks across species is described in Additional file 1, Figure S1.

### Conserved gene co-expression recovers known meiotic genes

**Figure 3:**
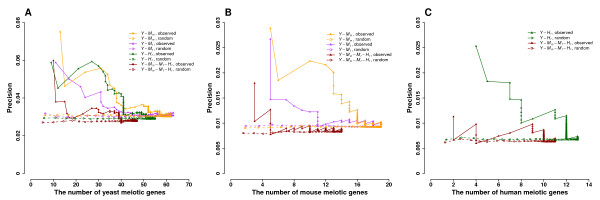
**Evaluation of conserved co-expression networks using known meiotic genes in yeast, mice, and humans**. Precision is the ratio of known meiotic genes (annotated by the meiosis term GO:0007126) to all metagenes. All metagene pairs in a co-expression network were sorted by P-value significance, and precisions were calculated in 100 pair increments. The random curves were derived from 100 trials of randomly permuting metagene pairs in each network. A. Four networks contain yeast genes. Among the 2,406 yeast genes that belong to metagenes, 72 are known meiotic genes. B. Three networks contain mouse genes. Among the 2,272 mouse genes that belong to metagenes, 19 are known meiotic genes. C. Two networks contain human genes. Among the 2,298 human genes that belong to metagenes, 13 are known meiotic genes.

We evaluated conserved co-expression networks using known meiotic genes annotated by gene ontology (GO:0007126) [20]. There are 72 yeast meiotic genes, 19 mouse meiotic genes, and 13 human meiotic genes among all metagenes, reflecting our limited knowledge of conserved meiotic processes in these three species. We plotted precision-coverage curves, which are the standard for determining method performance (Figure 3). Precision is defined as the ratio of known meiotic genes to all metagenes. Coverage is the number of known meiotic genes. All metagene pairs in each co-expression network were sorted by the significance of their P-values. We then calculated precision and coverage values in 100-pair increments to plot the curve. Yeast results show that a higher percentage of metagenes are meiotic in the top metagene pairs (Figure 3A). As we go down the list of metagene pairs by P-values, the precision of meiotic genes decreases; this was true for all four networks containing yeast genes. Interestingly, the top-100 metagene pairs in the network *Y-M_m _*contain a higher fraction of meiotic genes than the other three networks *Y-M_f_*, *Y-H_f_*, and *Y-M_m_-M_f_-H_f_*. This suggests that yeast meiosis might be more similar to the male mouse process than to the female process. Note that the low precision rate (y-axis) is not due to the computational method, but results from the very small number of known meiotic genes (x-axis) among all the genes in the networks.

When we used mouse and human meiotic genes to evaluate co-expression networks, we saw results similar to those in yeast (Figure 3). Top metagene pairs contain a higher fraction of meiotic genes. Meiotic gene enrichment declines when metagene pairs are ranked down the list. The network *Y-M_m _*is more enriched for mouse meiotic genes than *Y-M_f _*and *Y-M_m_-M_f_-H_f _*(Figure 3B), again suggesting the similarity between yeast meiosis and male meiosis.

We further evaluated enriched GO terms among the top-100 metagene pairs from four co-expression networks. We chose top-100 metagene pairs because they are highly enriched for meiotic genes (Figure 3). When using yeast genes to search for GO terms, we found that the meiosis term was significantly enriched in all four networks (P-values = 0.001 for *Y-M_m_*, 0.019 for *Y-M_f_*, 0.009 for *Y-H_f_*, 0.033 for *Y-M_m_-M_f_-H_f_*) (Table 2). Consistent with the precision-coverage results, this again indicates that our co-expression networks can efficiently identify known meiotic genes. Yeast genes participating in the cell cycle, in DNA metabolism, and in protein folding are also present in the co-expression networks. When using mouse genes to identify enriched GO terms among the top-100 metagene pairs, we identified many significant terms describing meiotic processes (Additional file 1, Table S1). Synapsis is the pairing of two homologous chromosomes that enables crossover during prophase. This term is enriched in both networks *Y-M_m _*and *Y-M_f _*(P-values = 0.022 for *Y-M_m_*, 0.001 for *Y-M_f_*,). Pachytene is the most significant GO term in the network *Y-M_m_-M_f_-H_f _*(P-value = 0.010). It is a stage of meiotic prophase characterized by synapsis and by the occurrence of crossover. Other enriched GO terms related to reproduction include female gamete generation, reciprocal meiotic recombination, DNA recombination, mismatch repair, microtubule-based movement, and so on. When using human genes to identify enriched GO terms among the top-100 metagene pairs (Additional file 1, Table S2), we again identified pachytene as a significant GO term in the network *Y-M_m_-M_f_-H_f _*(P-value = 0.010). We also found other enriched GO terms directly associated with reproduction, such as cell cycle, reciprocal meiotic recombination, DNA replication, meiosis, double-strand break repair, female gamete generation, and spermatogenesis.

**Table 2:** Significant GO SLIM terms enriched in yeast genes from the top-100 metagene pairs* in conserved co-expression networks

GO Term	GO Name	**Hypergeometric P-value**^**#**^
*Y-M_m_*		
GO:0007126	meiosis	0.001
GO:0007049	cell cycle	0.006
GO:0006950	response to stress	0.025
GO:0006997	nucleus organization	0.027

*Y-M_f_*		
GO:0007126	meiosis	0.019
GO:0007165	signal transduction	0.023
GO:0000746	conjugation	0.024
GO:0016044	membrane organization	0.030
GO:0007049	cell cycle	0.037
GO:0007114	cell budding	0.044

*Y-H_f_*		
GO:0006259	DNA metabolic process	0.001
GO:0006996	organelle organization	0.004
GO:0007126	meiosis	0.009
GO:0007010	cytoskeleton organization	0.010
GO:0006350	transcription	0.025
GO:0007049	cell cycle	0.035

*Y-M_m_-M_f_-H_f_*		
GO:0006457	protein folding	0.031
GO:0007126	meiosis	0.033

* Top metagene pairs are the pairs with the most significant P-values for co-expression across species.

^# ^Significant GO terms are defined by hypergeometric P-value < 0.05.

Next, we determined whether using conservation of co-expression between different species improved the identification of meiotic genes as compared to an alternative approach by using co-expression in a single species. To this end, we sorted metagene pairs by Pearson correlation coefficient in each microarray and plotted precision-coverage curves to evaluate co-expression in individual microarrays using known meiotic genes (Additional file 1, Figure S2). Interestingly, the conserved co-expression approach showed no improvement in predicting known yeast meiotic genes from top metagene pairs as compared to the co-expression approach in a single species (compare Figure 3A with Additional file 1, Figure S2A). However, the conserved co-expression approach displayed a higher enrichment of known mouse and human meiotic genes among top metagene pairs than the co-expression approach in individual species (compare Figure 3 with Additional file 1, Figure S2B-S2C). For example, only one known human meiotic gene was recovered in the top-100 metagene pairs from female human microarray by using the co-expression approach in individual species (Additional file 1, Figure S2C), while four were recovered from the top-100 metagene pairs by using the conserved co-expression between yeast and human studies (Figure 3C). We also identified enriched GO terms among the top-100 metagene pairs from individual microarrays (Additional file 1, Table S3). Similar to the results obtained from precision-coverage curves, the conserved co-expression approach showed no improvement for the yeast study in terms of enrichment of meiosis-related GO terms, but improved the enrichment of mammalian meiosis-related GO terms (compare Additional file 1, Table S3 with Table 2 and Additional file 1, Table S1 and Table S2). Finally, we compared the top-100 metagene pairs from the conserved co-expression approach with the top-100 metagene pairs from the co-expression approach in a single species, and found varied degrees of metagene pair overlap between the two sets, ranging from 0 to 26. In summary, the conserved co-expression approach can improve the identification of mammalian meiosis genes although the genes identified by this approach do not necessarily overlap with meiotic genes identified by the simple co-expression approach from individual species.

### Properties of conserved co-expression genes and gene pairs

**Figure 4:**
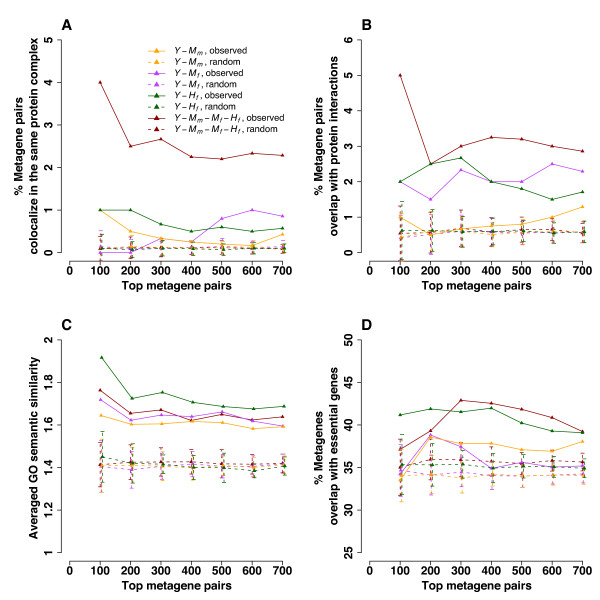
**Characterizations of metagene pairs and metagenes using yeast genomic information**. Top metagene pairs are the pairs with the most significant P-values for co-expression across species. The random curves with mean and standard deviation labeled were derived from 100 trials of randomly permuting metagene pairs in each network. A. Yeast metagene pairs co-localized in the same protein complex. The observed curve is significantly different from the randomized curve for all networks except *Y-M_f _*(Welch Two Sample t-test P < 0.05). B. Yeast metagene pairs overlapping with protein interactions. The observed curve is significantly different from the randomized curve for all four networks (Welch Two Sample t-test P < 0.05). C. Averaged semantic similarity of yeast metagene pairs calculated using GO sub-ontology Biological Process. The observed curve is significantly different from the randomized curve for all four networks (Welch Two Sample t-test P < 0.05). D. Yeast metagenes overlapping with essential genes. The observed curve is significantly different from the randomized curve for all four networks (Welch Two Sample t-test P < 0.05).

Because all four co-expression networks contain yeast genes, we took advantage of enriched yeast genomic information to investigate the properties of top ranked metagene pairs. We first examined whether significant co-expression links overlapped with protein interactions. Our results show that top co-expression metagene pairs in all four observed networks are more enriched for physical interactions as compared to those in randomized networks. This is evidenced by co-localization in the same MIPS protein complex [21] and by overlap with physical interactions retrieved from the BioGRID database [22] (Figure 4). In particular, top metagene pairs in the most conserved network, *Y-M_m_-M_f_-H_f_*, overlap with a higher fraction of physical interactions than those in the other three co-expression networks. The co-occurrence of protein interaction and expression correlation can suggest high-confidence functional associations of metagene pairs.

Next, we examined the functional associations of yeast metagene pairs using averaged GO semantic similarity in the sub-ontology Biological Process [23]. Biological Process implies a series of molecular events in which a gene participates. We found that the averaged semantic similarity of observed metagene pairs is greater than that of randomized pairs for all four co-expression networks (Figure 4C). The top-100 metagene pairs showed the tightest functional connection. This connection declined with increased inclusion of metagene pairs down the significant P-value list.

The yeast genome contains approximately 1,100 essential genes required for cell viability in the rich glucose medium [24]. This is equivalent to 18% of the total genes. The percentage of metagenes overlapping with essential genes was approximately 35% for randomized metagene pairs, suggesting evolutionarily conserved metagenes are enriched for essential genes (Figure 4D). Metagenes from the top co-expression pairs contained a slightly higher percentage of essential genes than those from randomized co-expression pairs. This indicates that a significant number of essential proteins may participate in the conserved meiotic process.

We also explored other yeast gene properties of the top metagene pairs in four co-expression networks. In a large-scale parallel phenotypic study, 261 sporulation-deficient genes and 102 sporulation-proficient genes were identified by screening 4,000 yeast deletion strains [25]. However, metagenes from the top co-expression pairs were not enriched for sporulation-proficient or deficient genes in all networks (data not shown). Inconsistent hits from global expression and deletion screens have been reported in many studies [4,24-26], and may be due to gene redundancy or indirect/non transcriptional responses. Synthetic lethality refers to a genetic interaction between two genes that cause cell death when they mutate concurrently, but neither by itself is lethal. The top metagene pairs were not enriched for synthetic lethal interactions (data not shown), although they were enriched for physical interactions. This is consistent with a previous finding that physical interactions imply function in a single pathway and are orthogonal to synthetic lethal interactions [27,28].

To examine mouse and human orthologs in co-expression networks, we compiled a list of mouse protein interactions from five databases (Biomolecular Interaction Network Database, Database of Interacting Proteins, Molecular INTeraction database, IntAct, and BioGrid) [22,29-32], and a list of human protein interactions from five databases (Human Protein Reference Database, Database of Interacting Proteins, Molecular INTeraction database, IntAct, and BioGrid) [22,30-33]. However, very few top-700 metagene pairs from any network overlapped with known human or mouse protein interactions. We believe this is due to the lack of coverage on protein-protein interactions in mammals because we did observe enriched yeast protein interactions among the top metagene pairs (Figure 4). The averaged GO semantic similarity of mouse and human genes showed a trend similar to that of the yeast genes (Figure 4C), with the top-100 pairs exhibiting the tightest functional connections (Additional file 1, Figure S3). Essential genes in humans [34,35] and mice [36] were not over-represented among the top metagene pairs (Additional file 1, Figure S4), which may also be related to mammalian data quality and coverage.

### Transcript abundance of conserved co-expression genes

**Figure 5:**
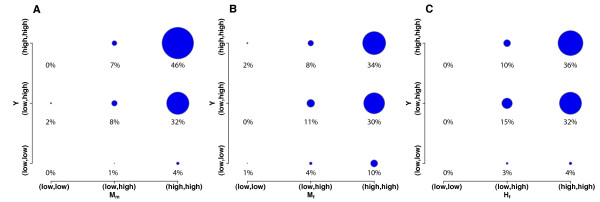
**Transcript abundance of genes in three two-species co-expression networks**. Top-100 metagene pairs with the most significant co-expression P-values were used for plotting the figure. The transcript abundance of genes from the metagene pairs was identified. The (low, low) means both genes have mRNA abundance below the median of genome-wide signal intensities, the (low, high) means one gene has mRNA abundance below the median of genome-wide signal intensities, with the other above the median, and the (high, high) means both genes have mRNA abundance above the median of genome-wide signal intensities. The size of circles corresponds to the number of metagene pairs. A. *Y-M_m _*network. B. *Y-M_f _*network. C. *Y-H_f _*network.

The gene co-expression was derived from Pearson correlation, which measures similarity of gene expression over time rather than absolute mRNA abundance. Therefore, we were interested in investigating the transcript abundance of metagene pairs in our networks (Figure 5). First, we identified maximal signal intensity for each gene over the course of prophase. Next, we ranked maximal signal intensities genome-wide to identify the median value. Any genes with maximal signal intensity above the median were defined as highly expressed genes, while the rest were defined as lowly expressed genes. Thus, we classified the top-100 metagene pairs in each two-species network as both highly expressed genes, both lowly expressed genes, or one highly expressed and one lowly expressed gene. We chose to analyze top-100 metagene pairs because they are highly enriched for meiotic genes and exhibit close functional connections (Figure 3, Figure 4).

Results show that most mouse and human orthologs of the top metagene pairs were highly expressed (Figure 5). This was demonstrated by the finding that 82% of the pairs in *Y-M_m _*were highly expressed in mouse postnatal testis, 74% of the pairs in *Y-M_f _*were highly expressed in mouse embryonic ovary, and 72% of the pairs in *Y-H_f _*were highly expressed in human fetal ovary. However, the transcript abundance of yeast genes did not follow the same trend as that in mammalian genes. In all three networks, approximately half of the yeast pairs had two highly expressed genes, whereas the other half of the pairs had one highly expressed and one lowly expressed gene. We observed that *Y-M_m _*has 46% metagene pairs that are consistently highly expressed in yeast and male mice (Figure 5A), whereas *Y-M_f _*and *Y-H_f _*have only 34% and 36% pairs consistently highly expressed between yeast and females (Figure 5). This again suggests that yeast meiosis might more closely resemble the male process than the female process.

To differentiate yeast metagene pairs with two highly expressed genes from those with one highly and one lowly expressed gene, we calculated the hypergeometric P-values of enriched GO terms for these two groups of yeast metagene pairs [20]. We found that the meiosis term (GO:0007126) was significantly enriched among metagene pairs with both genes highly expressed (P-values = 0.0003 for *Y-M_m_*, 0.05 for *Y-M_f_*, 0.005 for *Y-H_f_*). Indeed, meiosis is the most significant GO term for networks *Y-M_m _*and *Y-H_f_*. By contrast, the meiosis term was not enriched in the yeast metagene pairs with one highly expressed and one lowly expressed gene in all three networks. This suggests that most yeast meiotic genes are highly expressed during prophase. In fact, these highly expressed yeast meiotic genes in prophase are generally highly expressed throughout meiosis when we define the expression level based on the entire meiosis time course.

### Prediction of novel meiotic genes in prophase

We have demonstrated that conserved co-expression networks can successfully recover known meiotic genes (Figure 3). We have also shown that conserved co-expression pairs exhibit close functional connections, as evidenced by the GO annotation similarity and overlap with physical interactions (Figure 4). Therefore, we can predict novel meiotic genes through their co-expression linkages with known meiotic genes. More accurate predictions can be attained when co-expression linkages overlap with physical interactions or annotation similarity. These candidate meiotic genes can first be experimentally validated using a genetically tractable yeast system before being tested in a mammalian system.

**Figure 6:**
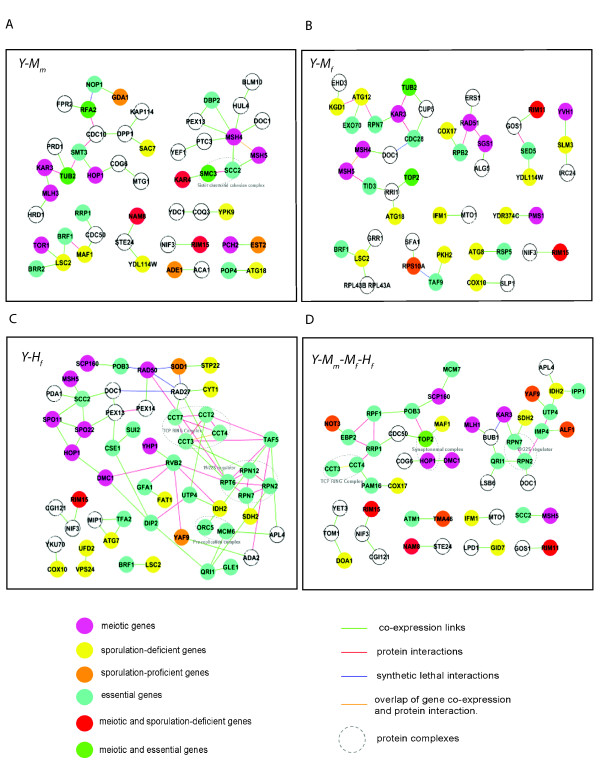
**Conserved gene modules in meiotic prophase**. Each conserved co-expression network was constructed by connecting the top-100 metagene pairs with the most significant P-values. Gene clusters containing either known yeast meiotic genes, sporulation-deficient genes, or sporulation-proficient genes were selected for display in the figure. Metagenes were labeled with yeast gene names. Metagenes and metagene connections were marked with different colors to represent information derived from yeast genomic datasets. A. *Y-M_m _*network. B. *Y-M_f _*network. C. *Y-H_f _*network. D. *Y-M_m_-M_f_-H_f _*network.

Four co-expression networks, *Y-M_m_*, *Y-M_f_*, *Y-H_f_*, and *Y-M_f_-M_m_-H_f_*, were constructed by linking the top-100 metagene pairs. We focus on top-100 metagene pairs because they are most enriched for meiotic genes and exhibit close functional connections (Figure 3, Figure 4). The top-100 metagene pairs in each network are listed in the Additional file 1, Table S4; many of these pairs are common to more than one network (Table 3). To identify meiotic prophase gene modules, we isolated gene clusters from networks containing yeast meiotic genes, sporulation-deficient genes, or sporulation-proficient genes (Figure 6) [20,25]. Because yeast is present in every network, we used yeast gene names to label the metagenes. Human and mouse orthologs of yeast genes can be easily mapped from metagenes (Table 4). Properties of yeast genes and their interactions were labeled onto these modules to facilitate interpretations, including essential genes, protein interactions, synthetic lethal interactions, and protein complexes [21,22,24].

**Table 3:** The top-100 metagene pairs* common to at least two conserved co-expression networks

Top-100 metagene pairs	***Y-M***_***m***_	***Y-M***_***f***_	***Y-H***_***f***_	***Y-M***_***m***_-***M***_***f***_-***H***_***f***_
*DED1-RTS2*	+	+	+	+
*CDC28-CUP5*	+	+	+	+
*NIF3-RIM15*	+	+	+	+

*RRI1-TID3*	+	+	+	
*PUT1-YOR289W*	+	+	+	
*CDC33-UNG1*	+	+	+	
*DUS3-MDJ1*	+	+	+	
*BRF1-LSC2*	+	+	+	
*BUD32-FET5*	+		+	+
*MSH5-SCC2*	+		+	+
*GET3-SRP102*		+	+	+

*DOC1-MSH4*	+	+		
*MSH4-MSH5*	+	+		
*PRE1-SMC6*	+	+		
*MLP1-YFR018C*	+	+		
*GOR1-YPL225W*	+	+		
*AGX1-YMR074C*	+		+	
*PYC1-SSU72*	+		+	
*MCM2-RRN3*		+	+	
*CCT5-YKE2*	+			+
*COG3-CYB5*	+			+
*COG6-HOP1*	+			+
*DPP1-KAP114*	+			+
*NAM8-STE24*	+			+
*PTC3-YEF1*	+			+
*RPL32-RPL37A*	+			+
*CDC50-RRP1*	+			+
*PUF6-RVB1*	+			+
*PNG1-SPT15*	+			+
*MTC5-TLG1*	+			+
*ASC1-RPP2B*		+		+
*APT1-ATP16*		+		+
*DIA4-SRP72*		+		+
*GOS1-RIM11*		+		+
*FOX2-HRD1*		+		+
*HRD1-RPN1*		+		+
*IFM1-MTO1*		+		+
*KAR3-RPN7*		+		+
*FMP32-LSM12*		+		+
*NFU1-RBG1*		+		+
*RPA135-VPS15*		+		+
*BZZ1-PRP16*			+	+
*DMC1-HOP1*			+	+
*IDH2-UTP4*			+	+
*MAK10-MAK5*			+	+
*CGI121-NIF3*			+	+
*CUL3-PCM1*			+	+
*QRI1-RPN2*			+	+
*RAD27-RPT6*			+	+
*RPN7-SDH2*			+	+
*POB3-SCP160*			+	+
*DDP1-SPB1*			+	+
*TLG1-TYW3*			+	+
*YBR241C-YNL155W*			+	+

* Top metagene pairs are the pairs with the most significant P-values for co-expression across species.

**Table 4:** Genes annotated by meiosis term (GO:0007126) from the top-100 metagene pairs* in conserved co-expression networks

	***Y-M***_***m***_	***Y-M***_***f***_	***Y-H***_***f***_	***Y-M***_***m***_-***M***_***f***_-***H***_***f***_
Yeast	*HOP1, KAR3, KAR4, MLH3, MSH4, MSH5, NAM8, PCH2, RFA2, RIM15, SMC3, TOR1, TUB2*	*KAR3, MSH4, MSH5, PMS1, RAD51, RIM11, RIM15, SGS1, TOP2, TUB2, YVH1*	*DMC1, HOP1, MSH5, RAD50, RIM15, SCP160, SPO11, SPO22, YHP1*	*DMC1, HOP1, KAR3, MLH1, MSH5, NAM8, RIM11, RIM15, SCP160, TOP2*

Mouse	*Mlh3, Msh4, Msh5, Smc3, Trip13*	*Msh4, Msh5, Pms2, Rad51, Tex15*	-	*Dmc1, Mlh1, Msh5*

Human	-	-	*DMC1, MSH5, RAD50, SPO11*	*DMC1, MSH5*

The time frame of microarray data used in our study covers meiotic prophase. Consistently, we observed the appearance of homologous recombination proteins and chromatin cohesion proteins in all four networks (Figure 6). These genes include *HOP1*, *DMC1*, *SPO11*, *SPO22*, *MSH4*, *MSH5*, and *SCC2*. The co-expression link between *HOP1 *and *DMC1 *was shown in networks *Y-H_f _*and *Y-M_m_-M_f_-H_f_*. *HOP1 *encodes for a meiosis-specific DNA binding protein and is required for homologous chromosome pairing. *DMC1 *encodes for a strand invasion protein, an essential component of the meiotic homologous recombination machinery [2]. The connection between *HOP1 *and *DMC1 *suggests the coordinated events of chromosome pairing and recombination. In the *Y-H_f _*network, *HOP1 *and *DMC1*were further linked to *SPO11*, *SPO22*, *MSH5*, and *SCC2*. These genes exhibit coherent functions and are located in the same cluster with both direct and indirect links. *SPO11 *initiates meiotic recombination by catalyzing the formation of double-strand breaks in DNA, while *SPO22 *is a meiosis-specific gene essential for chromosome pairing [1]. Msh4 and Msh5 are meiotic recombination proteins and form heterodimers [2]. They are connected by both co-expression and protein interaction in networks *Y-M_m _*and *Y-M_f_*. *MSH5 *also showed consistent co-expression links with *SCC2 *in networks *Y-M_m_*, *Y-H_f_*, and *Y-M_m_-M_f_-H_f_*. *SCC2 *is a cohesion loading factor involved in establishing sister chromatid cohesion during double strand break repair [37]. This indicates that regulation of sister chromatid cohesion is synchronized with recombination events during meiotic prophase. Interestingly, we observed that *DOC1 *is always associated with a cluster of *MSH4*, *MSH5*, and *SCC2 *in the three, two-species networks. Yeast Doc1 and its mammalian ortholog APC10 are a subunit of the anaphase promoting complex, a conserved ubiquitin ligase complex that degrades mitotic cyclins and anaphase inhibitory proteins, thereby triggering sister chromatid separation and exit from mitosis [38,39]. However, the involvement of *DOC1 *in the meiotic process has never been shown before. The co-expression link with recombination and cohesion proteins indicates that *DOC1 *might also function during meiotic prophase.

*NAM8 *is a component of the U1 snRNP protein involved in the formation of double strand breaks [40]. The *nam8 *deletion mutant is defective in sporulation [25]. A co-expression link between *NAM8 *and *STE24 *was observed in *Y-M_m _*and *Y-M_m_-M_f_-H_f_*. *STE24 *is a highly conserved zinc metalloprotease that functions in two steps of a-factor maturation in yeast [41]. Its human ortholog, *FACE1*, is highly expressed in testis and ovary [42]. The linkage between *NAM8 *and *STE24 *suggests a possible new role for *STE24 *in meiotic recombination. Furthermore, an uncharacterized ORF *YDL114W *is also connected to *STE24 *in *Y-M_m_*, and to the essential transporter gene *SED5 *[43] in *Y-M_f_*. *YDL114W *is predicted to have peptide transporter activity, possibly related to the fact that a-factor is a secreted peptide. Another uncharacterized ORF, *YDR374C*, exhibited a co-expression linkage with *PMS1 *in the network *Y-M_f_*. *PMS1 *encodes for an ATP-binding protein and is required for mismatch repair in meiosis [44]. This linkage predicts a possible role for *YDR374C *in meiotic recombination.

The general notion about meiosis conservation is that entry signaling has diverged substantially among species but mechanical components and enzymes are conserved [2]. For example, yeast cells initiate meiosis in a nutrient depleted sporulation medium, while mammalian germ cells initiate meiosis in response to extrinsic inducer retinoic acid [3,10,11]. However, our findings suggest potential players that might control the conserved meiotic entry process. We identified two genes, *RIM11 *and *RIM15*, from gene co-expression networks that are known to control meiotic entry in yeast. Both showed sporulation deficiency when knocked out [25], suggesting that the mammalian orthologs of *RIM11 *and *RIM15 *might play a similar role in governing meiotic entry. Rim11 is a protein kinase required for signal transduction during meiotic entry in yeast. It promotes the formation of the Ime1-Ume6 complex by phosphorylating Ime1 and Ume6 [45]. Glycogen synthase kinase-3b (*Gsk-3b*) is the mammalian ortholog of yeast *RIM11*. *Gsk-3b *participates in a variety of cell signaling events in addition to regulating glycogen synthesis [46]. It is expressed in both spermatocytes and Sertoli cells in mice and rats. Further, inhibition of GSK-3b has been shown to prevent DNA replication in cultured rat germ cells [47], supporting our prediction that *Gsk-3b *plays a role similar to that of its yeast ortholog, *RIM11*, in regulating meiotic entry in mammals. A co-expression link was observed between *RIM11 *and *GOS1 *in *Y-M_f _*and *Y-M_m_-M_f_-H_f_*. Yeast *GOS1 *and its mammalian ortholog, *Gosr1*, encode a SNARE protein that is involved in Golgi transport [48]. The connection between Golgi transport and meiotic entry is intriguing and previously has not been documented. *RIM15 *is a glucose-repressible protein kinase that has been identified as a regulator of *IME2*, a key gene that controls meiotic entry in yeast [49]. *Mastl *(microtubule-associated serine/threonine-protein kinase-like) is the mammalian ortholog of *RIM15*. *RIM15 *has a co-expression linkage to *NIF3 *in all four networks, suggesting that *NIF3 *may also be involved in regulation of meiotic entry. The function of yeast *NIF3 *is unknown [50]. Its mammalian ortholog *Nif3l *showed a ubiquitous expression pattern with encoded protein localized in the cytoplasm [51].

### Experimental validation of novel meiotic genes in prophase

Our computational research generated many candidate meiotic genes that function during meiotic prophase (Figure 6). Here we focus on validating candidates that show co-expression links with recombination genes, chromatin cohesion genes, and genes involved in meiotic entry in at least two networks. By taking this criterion, we experimentally tested five genes, *DOC1*, *STE24*, *COG6, GOS1*, and *NIF3*, plus *YDR374C*, an uncharacterized ORF co-expressed with *PMS1 *in only one network.

We conducted sporulation assays using yeast deletion strains of these genes (Table 5, Additional file 1, Figure S5). Homozygous diploid deletion strains have been systematically constructed for every yeast gene on the BY4743 genetic background [24]. At the end of the sporulation experiment, we calculated the percentages of cells completing meiosis I (binucleates+trinucleates+tetranucleates) and meiosis II (trinucleates+tetranucleates). Around 300 cells were analyzed for each strain [4,5]. Our positive control is the wild-type strain. Around 18% of the wild-type cells in the population went through meiosis I and II and then sporulated. This sporulation efficiency is consistent with previous observation on the BY4743 wild-type strain [24]. Our negative controls are deletion strains of *rim11 *and *rim15*; both are required for signal transduction during meiotic entry in yeast [25]. We observed that the sporulation process was completely blocked for these two deletion strains. Next, we tested the sporulation efficiency of the six candidate meiotic genes. We found that all of them showed sporulation defects, equivalent to a 100% validation rate. Three that are predicted to be involved in meiotic recombination (*DOC1*, *STE24*, and *YDR374C*) exhibited three- to six-fold reductions in sporulation efficiency. In particular, no tetranucleate cell was observed in the *doc1 *mutant. *COG6*, with a co-expression link to *HOP1*, showed a 1.4-fold decrease in sporulation efficiency. *GOS1 *and *NIF3 *exhibited co-expression linkages with *RIM11 *and *RIM15*, respectively. Deletion mutants of *gos1 *and *nif3 *show 1.5- to 2.5- fold decreases in sporulation efficiency. The above experimental verification demonstrates that our conserved co-expression network provides a powerful tool for discovering novel meiotic genes. Mammalian orthologs of these genes can be further evaluated using a mouse system.

**Table 5:** Validation of predicted meiotic genes by sporulation assay

	Strain	Gene	% cells completing meiosis I*	**% cells completing meiosis II**^**#**^
Positive control	BY4743	-	18.3%	17.7%

Negative control	BY4743 *rim11*	*RIM11*	0%	0%
	BY4743 *rim15*	*RIM15*	0%	0%

Predicted meiotic genes	BY4743 *doc1*	*DOC1*	3.2%	0.4%
	BY4743 *ste24*	*STE24*	5.3%	4.7%
	BY4743 ydr374c	*YDR374C*	4.5%	4.5%
	BY4743 *cog6*	*COG6*	13.6%	13.0%
	BY4743 *gos1*	*GOS1*	8.2%	7.2%
	BY4743 *nif3*	*NIF3*	12.4%	10.9%

* Percentage of binucleates, trinucleates, and tetranucleates among 300 cells for each strain.

^# ^Percentage of trinucleates and tetranucleates among 300 cells for each strain.

## Discussion

Meiotic prophase is a critical stage in determining reproductive success, as errors in meiotic initiation and recombination can lead to chromosome mis-segregation. In fact, defects in meiotic chromosome segregation are the leading cause of miscarriages and one of the leading causes of birth defects in humans [1]. Identification of germ-cell-specific meiotic genes in multicellular organisms is a complex task because gonads contain distinct types of cells, of which only a fraction are germ cells. Because human gonads are an intractable experimental system, human meiosis has mainly been investigated using traditional *in vivo *mouse genetics, which is time-consuming and difficult to scale up. Only a limited number of mammalian meiotic genes have been characterized; for this reason, it is important to be able to predict novel meiotic genes in mammals.

In this study, we assembled cross-species and cross-sex whole-genome expression profiles on meiotic prophase in yeast, mouse embryonic ovary, mouse postnatal testis, and human fetal ovary. We found significant enrichment of known meiotic genes in co-expression networks, suggesting the feasibility of our approach for inferring conserved meiotic genes in multiple species and between sexes. Indeed, conservation of co-expression between species improved the identification of mammalian meiosis genes. We further characterized co-expression pairs and demonstrated that they are functionally related. Most top-ranked co-expression genes are highly expressed, particularly in mammals. From co-expression networks, we identified genes important to the meiotic process in both yeast and mammals. Our results show that major recombination and cohesion proteins are conserved across species. We also identified mammalian orthologs of yeast meiotic genes *RIM11 *and *RIM15 *as candidates that might regulate meiotic entry in mammals. Co-expression links enabled us to infer roles for genes not previously found to function during the meiotic process. We experimentally validated six predicted meiotic genes using a genetically tractable yeast system, all of which exhibited sporulation defects. The mammalian orthologs of these new meiotic genes can be further tested using a mouse system. In contrast to the simple clustering of meiosis expression profiles which could pinpoint many meiotic gene candidates [6-9,15,16], our method quantifies conserved co-expression with P-values in four networks, which allows us to prioritize candidate genes and interactions for experimental testing.

We focused on validating candidate genes showing co-expression links with known meiotic genes. This criterion is likely to yield a high validation rate. Our future direction will include testing conserved co-expression metagene pairs common to multiple networks. This would very likely increase the list of candidate genes that function during conserved meiotic process. In our study, we used microarray profiles of testes and ovaries that closely represent *in vivo *germ cell gene expression. A complementary approach is to use expression data from isolated germ cells; such data is only available in males [8,15]. Although sorted germ cell samples removed most somatic cells, their expression patterns may have changed dramatically from *in vivo *status. The microarray studies use in our analysis were not particularly designed to capture transcriptional changes in meiotic prophase except for the data on human fetal ovary [6]. The yeast experiments covered the entire sporulation process [5], while the mouse experiments captured the developmental process of the murine embryonic gonad and the first wave of spermatogenesis in postnatal testis [8,9]. Therefore, only limited time points were included to describe meiotic prophase. Our approach will likely become more valuable for the identification of novel genes when expression data are available to capture detailed transcriptional changes during meiotic prophase. This will enable us to better understand the genetic controls that regulate meiotic entry and progression in this critical developmental stage.

## Conclusions

We constructed conserved co-expression networks for meiotic prophase by integrating cross-species and cross-sex expression profiles from budding yeast, mouse, and human. The co-expression links in the networks confirmed known meiotic genes and identified several novel genes that might be critical players in meiosis in multiple species. Indeed, the conserved co-expression approach improved the identification of mammalian meiotic genes. Six candidate genes were subsequently validated in the yeast and all showed sporulation defects. These results suggest our approach is highly efficient to identify evolutionarily conserved gene modules and novel genes in meiotic prophase.

## Methods

### Metagene construction

Pairwise ortholog groups of yeast, mouse, and human were downloaded from Inparanoid, a database of eukaryotic orthologs [52]. Only seed orthologs found through a reciprocal best match between two genomes were kept for metagene construction. Three types of metagenes were obtained by identifying orthologs conserved either across all three species (YMH) or only between two species (YM, YH) (Table 1). Three metagene types are mutually exclusive. Most metagenes contain a single gene from each organism. Each gene was assigned to only one metagene.

### Microarray expression profiles

Four time-series microarray studies were selected to investigate global gene expression of meiotic prophase in yeast, mouse postnatal testis (GSE12769), mouse embryonic ovary (GSE6916), and human fetal ovary (GSE15431) [5,6,8,9]. These experiments all used the Affymetrix microarray platform. For the yeast experiment, gene expression was monitored using aliquots of SK1 cells at 0, 1, 2, 3, 4, 6, 8 and 10 hours after transfer of cells to sporulation medium [5]. For microarray profiling of the first wave of spermatogenesis, duplicate testis samples were obtained from postnatal mice at ages of 0, 3, 6, 8, 10, 14, 18, 20, 30, 35, and 56 days postpartum [8]. For female mice, duplicate samples of embryonic ovaries were collected at 11.5, 12.5, 14.5, 16.5, and 18.5 days of postcoitum [9]. For female humans, ovaries from fetuses at 9.1, 9.6, 11, 12, 12.9, 13.6, 13.9, 14.4, 16.1, 16.4, 16.9, 17.1, and 18.1 weeks of gestation were obtained; each time-point was represented by one fetus sample except there were three samples at 9.6 weeks, two at 13.6 weeks and two at 16.9 weeks [6]. Although the human female microarray captures the timeframe of meiotic prophase, the yeast and male mouse microarrays cover the entire meiosis, and the female mouse experiment was designed to capture the development of the murine embryonic ovary. To consistently identify changes in gene expression during meiotic prophase, only time points within meiotic prophase were considered in our analysis (0-4 hours for yeast, 6-14 days postpartum for mouse postnatal testis, 11-14 days postcoitum for mouse embryonic ovary, 9-18 weeks gestation for human fetal ovary).

Microarray data were normalized to the mean or median of each array, as described in the original papers [5,6,8,9]. For human and mouse experiments, unique probes that map to the same gene were averaged to obtain the gene signal intensity. If unique probes do not exist for a gene, we averaged signal intensity of probes with a "_a" suffix. Duplicate samples at each time point were averaged. The maximal signal intensity over the course of prophase was identified for each gene in each microarray. We used this signal intensity to define gene expression levels. The top 90% of highly expressed genes in each array were used to calculate Pearson correlations of gene pairs, while the bottom 10% of lowly expressed genes were removed from further study. This prevented the introduction of very lowly expressed genes into the conserved co-expression network. We did this because Pearson correlations measure similarity of gene expression rather than absolute levels, and lowly expressed gene profiles are often dominated by noise.

### Conserved gene co-expression networks

Pearson correlations, $r_{xy}=\frac{\sum_{i=1}^{n}(x_{i}-\bar{x})(y_{i}-\bar{y})}{(n-1)S_{x}S_{y}}$, were calculated for gene pairs belonging to metagenes across the prophase time points in each microarray. Here *x *and *y *are expression data vectors of length *n *for two genes, $\bar{x}$ and $\bar{y}$ are means, and *s_x _*and *s_y _*are standard deviations. If a metagene contains more than one gene in a species, Pearson correlations for metagene pairs were computed by averaging multiple gene-gene Pearson correlations. Otherwise, Pearson correlations for metagene pairs are the same as correlations for gene pairs. Metagene pairs in the same species were ranked by their Pearson correlations. Then each metagene pair was associated with a rank ratio, *r*, which was the rank divided by the total number of metagene pairs in a species *s*. We computed the P-value for each metagene pair across a total of *n *species from the joint cumulative distribution of an *n-*dimensional order statistics [19]: $P\left(r_{1}r_{2},...,r_{n}\right)=n!\int_{0}^{r_{1}}\int_{s_{1}}^{r_{2}}...\int_{s_{n-1}}^{r_{n}}ds_{1}ds_{2}...ds_{n},$, where *r*_1 _<*r*_2 _... < r_*n*_. This P-value quantifies the evolutionarily conserved co-expression of metagene pairs. Metagene pairs with P-values greater than a threshold can be connected to form conserved co-expression networks.

### Hierarchical clustering for identifying conserved co-expression

An alternative to our approach is to cluster Pearson correlation matrix to identify conserved co-expression. Pearson correlation of metagene pairs was calculated for each species. The pairwise correlation matrices for different species can be merged into a consensus correlation matrix by taking the minimum correlation for each metagene pair. The resulting consensus matrix was converted into a dissimilarity matrix and subjected to average linkage hierarchical clustering algorithms. The dynamic tree cut method was applied to define branches as co-expression modules (minClusterSize = 40) [53]. Order statistics and hierarchical clustering perform similarly in identifying known meiotic genes (Additional file 1, Table S5). The advantage of order statistics over hierarchical clustering is that it defines pairwise gene co-expression thus can prioritize genes for experimental testing.

### GO term enrichment

Significantly shared GO terms are used to describe common functions of a query gene set. To determine whether any GO terms are enriched in a query gene list at a frequency greater than that would be expected by chance, we calculated the probability from a hypergeometric distribution: $p\left(x\geq k\right)=\sum_{x=k}^{\min\left(m,n\right)}C\left(m,x\right)C\left(t-m,n-x\right)/C\left(t,n\right)$ where *C(j, k) *is the combinatorial factor *j!/k!(j-k)!*. In this equation, *t *is the total number of genes in a network, *n *is the number of genes in the network that are annotated by a GO term, *m *is the number of genes in a query list (for example, genes in top-100 metagene pairs), and *k *is the number of genes in that list which are annotated by the GO term.

### GO semantic similarity

The method to calculate GO semantic similarity was described in the reference [23]. Specifically, each node in the GO tree is associated with a probability, *p(c)*, representing the chance a concept occurs on the node or any of its children. Thus the probability increases as we move up toward the root of GO tree, where the probability is 1. To calculate the semantic similarity for a pair of genes, the minimal *p(c) *of parental nodes shared by two genes will be identified. The similarity score is defined as the negative log10 of the minimal *p(c)*. Because each gene pair is associated with a semantic similarity score, the averaged GO semantic similarity is the mean of similarity scores of a group of gene pairs. The averaged GO semantic similarity can be used to quantify the overall functional association of a group of gene pairs.

### Yeast sporulation assays

Wild-type yeast strain BY4743 and homozygous diploid deletion strains on the BY4743 background (isogenic to the strain S288c) were patched onto a GNA pre-sporulation plate. The GNA plate was incubated at 30°C overnight. Cells were transferred to the sporulation medium and incubated on a shaker at 25°C for 5 days [24]. Approximately 0.1OD cells were fixed with 70% ethanol for 30 minutes. Samples were washed with PBS twice, and then stained with 2uM Hoechst 33342 (H1399 Invitrogen) at room temperature for 30 minutes. Cells containing tetranucleate, trinucleate, binucleate, and mononucleate were counted from a total of 300 cells for each strain using a Zeiss 510 Meta multiphoton confocal microscope.

## Authors' contributions

YL and PY carried out the computational studies. KL carried out the sporulation assays. ND helped with the order statistics. PY conceived of and supervised the project. PY wrote the paper. All authors read and approved the final manuscript.

## Supplementary Material

Additional file 1 **Supplementary figures and tables**. This file includes five supplementary figures and five supplementary tables. Click here for file
