# Supplementary material for: A yeast's eye view of mammalian reproduction: cross-species gene co-expression in meiotic prophase
Source: BMC Syst Biol. 2010 Sep 6;4:125. doi: 10.1186/1752-0509-4-125 (PMC2944139; doi:10.1186/1752-0509-4-125)
Supplement: Additional file 1 — Supplementary figures and tables. This file includes five supplementary figures and five supplementary tables. [file 1752-0509-4-125-S1.PDF]

Additional file 1

A yeast's eye view of mammalian reproduction: cross-species gene co-expression in meiotic prophase

Yunfei Li[1,3], Ka-sum Lam[1], Nairanjana Dasgupta[3], Ping Ye[1,2,§]

[1]School of Molecular Biosciences, Washington State University, PO Box 647520, Pullman, WA 99164, USA

[2]Center for Reproductive Biology, Washington State University, Pullman, WA 99164, USA

[3]Department of Statistics, Washington State University, Pullman, WA 99164, USA

[§]Correspondence author

Metagene pair	Yeast microarray		Male mouse microarray		$Y\text{-}M_m$ network
	Pearson correlation	Rank ratio	Pearson correlation	Rank ratio	Order statistics P-value
A-B	0.9	1/3	0.5	2/3	P(1/3,2/3)=0.33
A-C	0.8	2/3	0.6	1/3	P(1/3,2/3)=0.33
B-C	0.7	3/3	0.4	3/3	P(3/3,3/3)=1

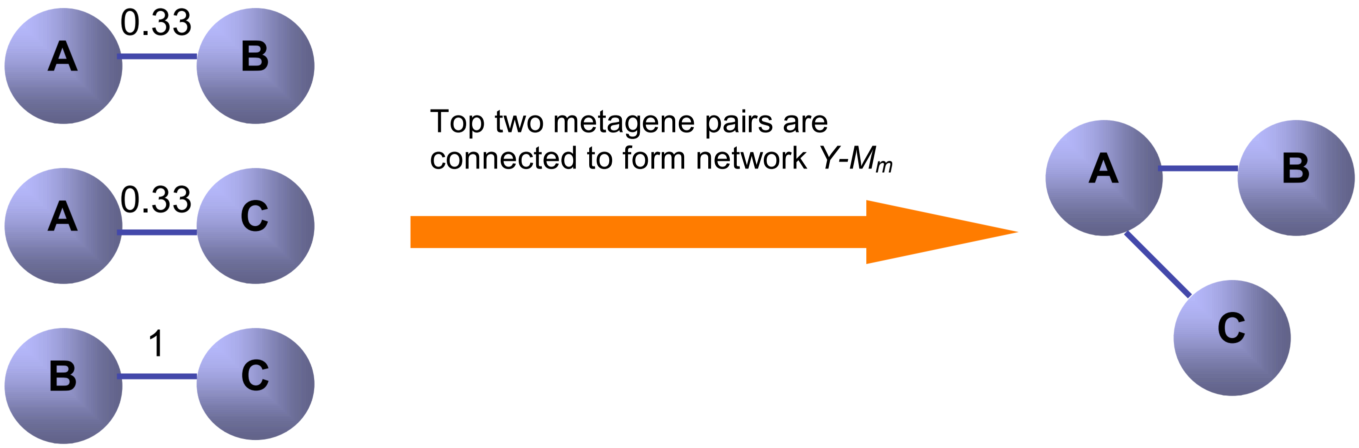

Figure S1 - A toy example for constructing conserved gene co-expression networks

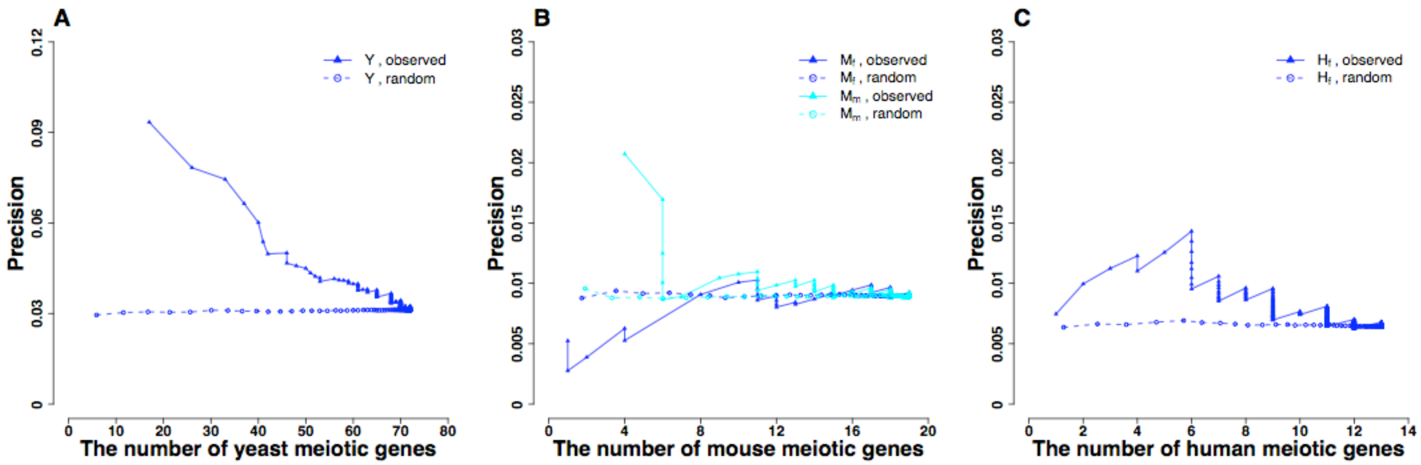

Figure S2 – Evaluation of gene co-expression from individual microarray studies using known meiotic genes in yeast, mice, and humans

Precision is the ratio of known meiotic genes (annotated by the meiosis term GO:0007126) to all metagenes. All metagene pairs in each microarray study were sorted by Pearson correlation coefficients, and precisions were calculated in 100 pair increments. The random curves were derived from 100 trials of randomly permuting metagene pairs. A. Co-expression pairs from the yeast microarray study. B. Co-expression pairs from the male mouse and female mouse microarray studies. C. Co-expression pairs from the female human microarray study.

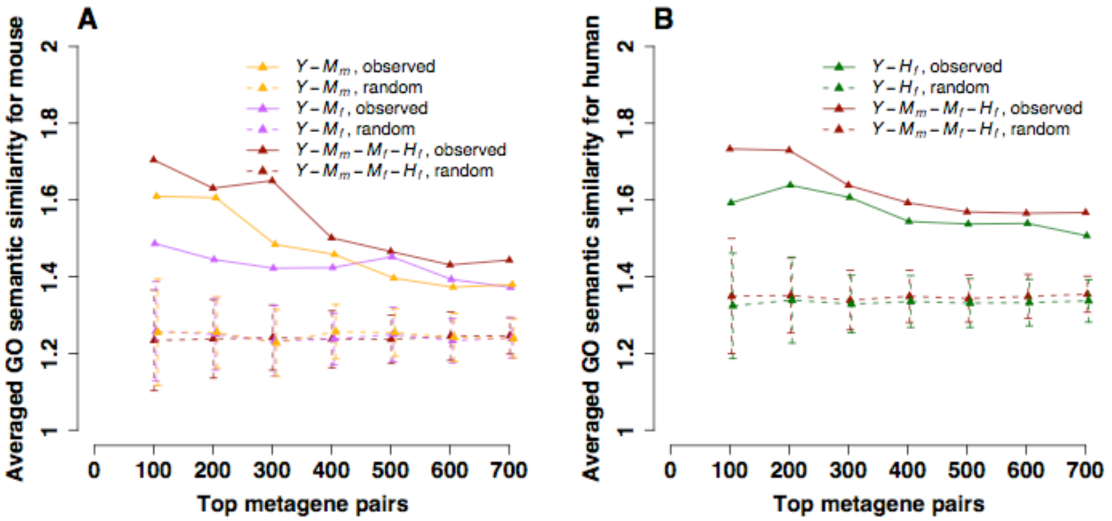

Figure S3 – Averaged semantic similarity of mouse and human metagene pairs calculated using GO sub-ontology Biological Process

Top metagene pairs are the pairs with the most significant P-values for co-expression across species. The random curves with mean and standard deviation labeled were derived from 100 trials of randomly permuting metagene pairs in each network. A. Mouse genes. B. Human genes.

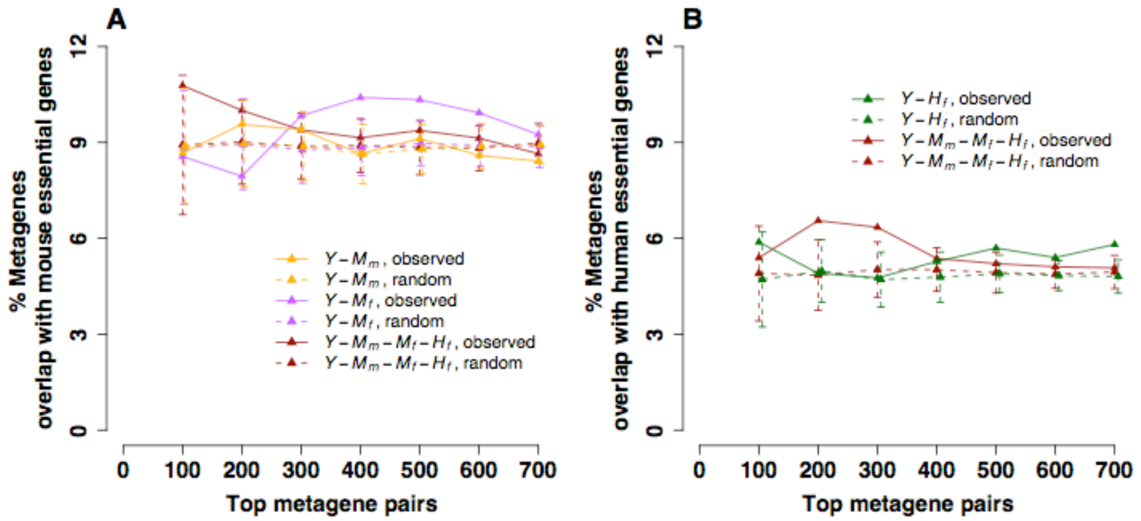

Figure S4 - Mouse and human essential genes are not enriched among top metagene pairs
Top metagene pairs are the pairs with the most significant P-values for co-expression across species. The random curves with mean and standard deviation labeled were derived from 100 trials of randomly permuting metagene pairs in each network. A. Mouse essential genes. B. Human essential genes.

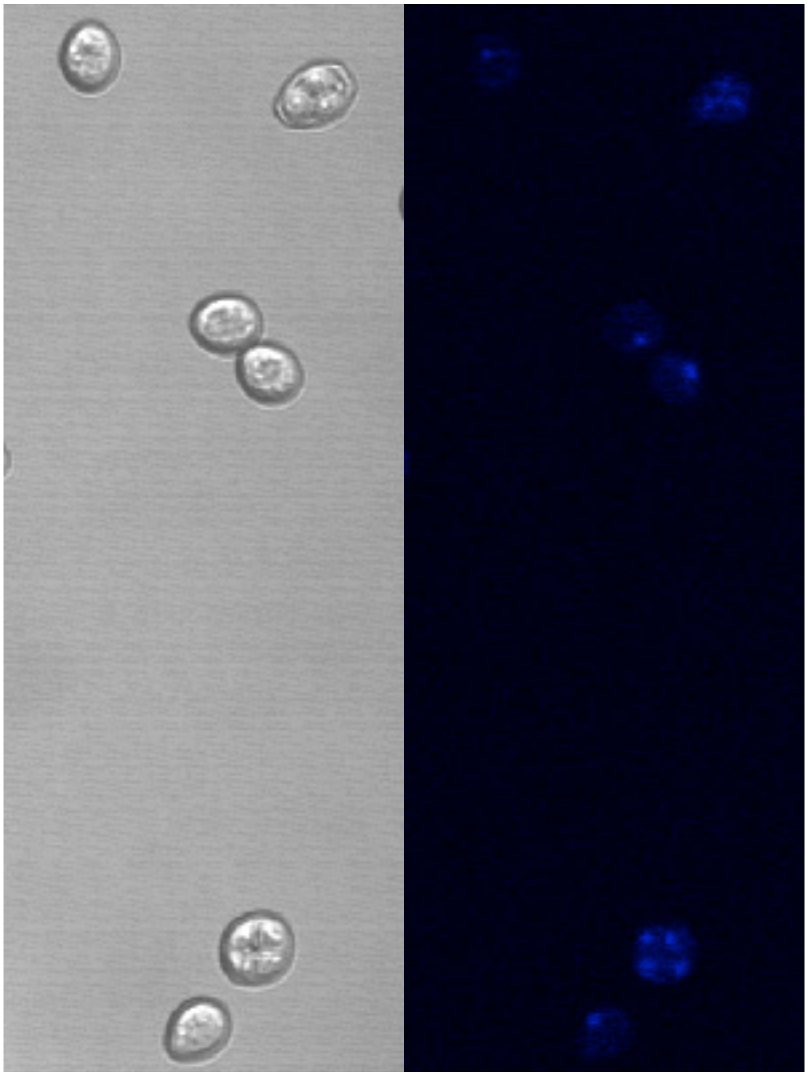

Figure S5 - Microscopy of sporulated yeast cells
The left panel shows light microscopy, and the right panel shows DNA staining with Hoechst in fluorescence microscopy.

Table S1 - Significant GO terms enriched in mouse genes from the top-100 metagene pairs* in conserved co-expression networks

GO Term	GO Name[#]	Hypergeometric P-value[&]
$Y\text{-}M_m$		
GO:0007018	*microtubule-based movement*	0.005
GO:0060152	*microtubule-based peroxisome localization*	0.008
GO:0007292	*female gamete generation*	0.008
GO:0007144	*female meiosis I*	0.008
GO:0007129	*synapsis*	0.022
GO:0032088	negative regulation of NF-kappaB	0.022
GO:0032312	regulation of ARF GTPase activity	0.022
GO:0018107	peptidyl-threonine phosphorylatio	0.022
GO:0006298	*mismatch repair*	0.027
GO:0007067	mitosis	0.031
GO:0006310	*DNA recombination*	0.037
GO:0006413	translational initiation	0.046
$Y\text{-}M_f$		
GO:0007129	*synapsis*	0.001
GO:0007292	*female gamete generation*	0.009
GO:0006310	*DNA recombination*	0.009
GO:0007242	intracellular signaling cascade	0.010
GO:0007017	*microtubule-based process*	0.013
GO:0006352	transcription initiation	0.013
GO:0006470	protein amino acid dephosphorylation	0.020
GO:0006298	*mismatch repair*	0.031
GO:0008150	biological_process	0.034
GO:0007018	*microtubule-based movement*	0.044
GO:0006611	protein export from nucleus	0.046
GO:0001541	*ovarian follicle development*	0.046
$Y\text{-}M_m\text{-}M_f\text{-}H_f$		
GO:0000239	*pachytene*	0.010
GO:0009790	*embryonic development*	0.016
GO:0006364	rRNA processing	0.020
GO:0007131	*reciprocal meiotic recombination*	0.028
GO:0032088	negative regulation of NF-kappaB	0.028
GO:0042176	regulation of protein catabolic process	0.028
GO:0008217	regulation of blood pressure	0.028
GO:0006457	protein folding	0.046

* Top metagene pairs are the pairs with the most significant P-values for co-expression across species.

[#] GO names related with meiosis are in italic.

[&] Significant GO terms are defined by hypergeometric P-value < 0.05.

Table S2 - Significant GO terms enriched in human genes from the top-100 metagene pairs* in conserved co-expression networks

GO Term	GO Name#	Hypergeometric P-value&
Y-H_f		
GO:0006366	transcription from RNA polymerase II promoter	0.001
GO:0007049	*cell cycle*	0.005
GO:0007131	*reciprocal meiotic recombination*	0.006
GO:0006260	*DNA replication*	0.006
GO:0030163	protein catabolic process	0.006
GO:0006457	protein folding	0.006
GO:0048844	artery morphogenesis	0.006
GO:0006048	UDP-N-acetylglucosamine biosynthetic process	0.006
GO:0006270	*DNA replication initiation*	0.009
GO:0007126	*meiosis*	0.012
GO:0006302	*double-strand break repair*	0.014
GO:0045893	positive regulation of transcription, DNA-dependent	0.016
GO:0006259	DNA metabolic process	0.018
GO:0007292	*female gamete generation*	0.018
GO:0007369	gastrulation	0.018
GO:0006264	mitochondrial DNA replication	0.018
GO:0000723	telomere maintenance	0.018
GO:0030512	negative regulation of transforming growth factor beta receptor signaling pathway	0.018
GO:0006915	apoptosis	0.025
GO:0006468	protein amino acid phosphorylation	0.025
GO:0006749	glutathione metabolic process	0.030
GO:0016558	protein import into peroxisome matrix	0.035
GO:0008643	carbohydrate transport	0.035
GO:0007569	cell aging	0.035
GO:0030308	negative regulation of cell growth	0.035
GO:0031145	anaphase-promoting complex-dependent proteasomal ubiquitin-dependent protein catabolic process	0.037
GO:0006417	regulation of translation	0.041
GO:0051436	negative regulation of ubiquitin-protein ligase activity during mitotic cell cycle	0.042
Y-M_m-M_f-H_f		
GO:0001701	*in utero embryonic development*	0.008
GO:0000239	*pachytene*	0.010
GO:0006048	UDP-N-acetylglucosamine biosynthetic process	0.010
GO:0022904	respiratory electron transport chain	0.010
GO:0007568	aging	0.015
GO:0007283	*spermatogenesis*	0.016
GO:0007067	mitosis	0.022
GO:0006468	protein amino acid phosphorylation	0.027

GO:0007369	gastrulation	0.027
GO:0050790	regulation of catalytic activity	0.027

* Top metagene pairs are the pairs with the most significant P-values for co-expression across species.
[#] GO names related with meiosis are in italic.
[&] Significant GO terms are defined by hypergeometric P-value < 0.05.

Table S3 - Significant GO terms enriched in genes from the top-100 metagene pairs* in individual microarray studies

GO Term	GO Name[#]	Hypergeometric P-value[&]
Y		
GO:0007126	*meiosis*	1.48×10^{-5}
GO:0007049	*cell cycle*	2.60×10^{-4}
GO:0006259	DNA metabolic process	0.012
GO:0007059	chromosome segregation	0.027
GO:0006950	response to stress	0.049
M_m		
GO:0006457	protein folding	0.004
GO:0006048	UDP-N-acetylglucosamine biosynthetic process	0.007
GO:0000045	autophagic vacuole formation	0.020
GO:0018107	peptidyl-threonine phosphorylation	0.020
GO:0006744	ubiquinone biosynthetic process	0.025
GO:0006096	glycolysis	0.033
GO:0006098	pentose-phosphate shunt	0.038
GO:0019221	cytokine-mediated signaling pathway	0.038
M_f		
GO:0032259	methylation	0.002
GO:0000209	protein polyubiquitination	0.007
GO:0000375	RNA splicing, via transesterification reactions	0.007
GO:0006491	N-glycan processing	0.007
GO:0043039	tRNA aminoacylation	0.020
GO:0006476	protein amino acid deacetylation	0.020
GO:0009790	*embryonic development*	0.024
GO:0031214	biomineral formation	0.038
GO:0006479	protein amino acid methylation	0.038
GO:0016070	RNA metabolic process	0.038
GO:0006526	arginine biosynthetic process	0.038
H_f		
GO:0007283	*spermatogenesis*	3.80×10^{-4}
GO:0000712	*resolution of meiotic joint molecules as recombinants*	0.003
GO:0007060	*male meiosis chromosome segregation*	0.003
GO:0006266	DNA ligation	0.003
GO:0006048	UDP-N-acetylglucosamine biosynthetic process	0.003
GO:0006897	Endocytosis	0.005
GO:0045893	positive regulation of transcription, DNA-dependent	0.009
GO:0006378	mRNA polyadenylation	0.010
GO:0006302	*double-strand break repair*	0.012
GO:0007067	Mitosis	0.012
GO:0006955	immune response	0.017
GO:0045190	isotype switching	0.019
GO:0007369	Gastrulation	0.019
GO:0046034	ATP metabolic process	0.019
GO:0016568	chromatin modification	0.020
GO:0006303	*double-strand break repair via nonhomologous end joining*	0.030
GO:0043966	histone H3 acetylation	0.043

GO:0006096	glycolysis	0.044
GO:0006364	rRNA processing	0.044

* Top metagene pairs are the pairs with the highest Pearson correlations for co-expression in a single microarray study.

[#] GO names related with meiosis are in italic.

[&] Significant GO terms are defined by hypergeometric P-value < 0.05.

Table S4 - The top-100 metagene pairs* in four conserved co-expression networks

Rank	Top-100 metagene pairs	P-value	Unique to the network
$Y\text{-}M_m$			
1	*PUF6-RVB1*	1.83E-08	N
2	*DED1-RTS2*	1.00E-07	N
3	*CCT3-GPI11*	1.22E-07	Y
4	*NPA3-PRE8*	2.82E-07	Y
5	*COG3-CYB5*	3.19E-07	N
6	*PRD1-TUB2*	3.68E-07	Y
7	*HUL4-MSH4*	3.90E-07	Y
8	*MLP1-YFR018C*	4.06E-07	N
9	*PHR1-RKR1*	4.28E-07	Y
10	*DOC1-MSH4*	4.70E-07	N
11	*MSH5-SCC2*	4.79E-07	N
12	*DBP2-PEX13*	5.33E-07	Y
13	*ACA1-ADE1*	6.03E-07	Y
14	*CDC28-CUP5*	6.07E-07	N
15	*CAT5-TMA22*	6.70E-07	Y
16	*MTC5-TLG1*	6.79E-07	N
17	*MSS4-NPL4*	6.80E-07	Y
18	*BRF1-LSC2*	7.12E-07	N
19	*COG6-HOP1*	8.88E-07	N
20	*NAM8-STE24*	1.06E-06	N
21	*KIP1-TSA1*	1.06E-06	Y
22	*KIP3-NSP1*	1.11E-06	Y
23	*CDC50-RRP1*	1.17E-06	N
24	*GCS1-UBC13*	1.19E-06	Y
25	*EST2-PCH2*	1.24E-06	Y
26	*POT1-URA2*	1.28E-06	Y
27	*PBS2-STP2*	1.40E-06	Y
28	*NIF3-RIM15*	1.41E-06	N
29	*GLE2-LCB2*	1.52E-06	Y
30	*AGE2-ERP2*	1.54E-06	Y
31	*KAR2-PTC7*	1.55E-06	Y
32	*RPL32-RPL37A*	1.56E-06	N
33	*OST1-SEC59*	1.75E-06	Y
34	*RPN6-SED5*	1.92E-06	Y
35	*HRD1-MLH3*	1.97E-06	Y
36	*DBP2-MSH4*	2.09E-06	Y
37	*DPP1-SAC7*	2.13E-06	Y
38	*PYC1-SSU72*	2.18E-06	N
39	*MSH4-SCC2*	2.24E-06	Y
40	*PCM1-PEX1*	2.36E-06	Y
41	*AGX1-YMR074C*	2.46E-06	N
42	*FPR2-RFA2*	2.59E-06	Y
43	*DPP1-KAP114*	2.65E-06	N
44	*FRA1-SPE2*	2.67E-06	Y
45	*LSC2-TOR1*	2.74E-06	Y
46	*SLP1-YPL191C*	2.80E-06	Y

47	*SMC4-YNL092W*	2.88E-06	Y
48	*CCT5-YKE2*	2.95E-06	N
49	*CDC50-MAF1*	2.95E-06	Y
50	*SPT15-SWC4*	3.05E-06	Y
51	*KAR4-SMC3*	3.06E-06	Y
52	*GDA1-NOP1*	3.13E-06	Y
53	*COG6-MTG1*	3.36E-06	Y
54	*CDC10-DPP1*	3.55E-06	Y
55	*ERV14-YDR341C*	3.60E-06	Y
56	*ENT3-YKU70*	3.63E-06	Y
57	*PRE1-SMC6*	3.77E-06	N
58	*STE24-YDL114W*	3.97E-06	Y
59	*MSH4-MSH5*	4.05E-06	N
60	*IMP1-PTP3*	4.05E-06	Y
61	*CAB4-NTF2*	4.06E-06	Y
62	*MSH4-PTC3*	4.11E-06	Y
63	*CEX1-VPS53*	4.13E-06	Y
64	*HOP1-SMT3*	4.15E-06	Y
65	*MSH4-PEX13*	4.15E-06	Y
66	*PAM16-YDL119C*	4.38E-06	Y
67	*GOR1-YPL225W*	4.39E-06	N
68	*BLM10-HUL4*	4.40E-06	Y
69	*RRI1-TID3*	4.45E-06	N
70	*HAP3-RPL13B*	4.50E-06	Y
71	*PNG1-SPT15*	4.59E-06	N
72	*ERG27-GSH1*	4.64E-06	Y
73	*CAB2-RCL1*	4.77E-06	Y
74	*PUT1-YOR289W*	4.82E-06	N
75	*AAD4-NUC1*	4.86E-06	Y
76	*SUA7-VMA13*	4.86E-06	Y
77	*ALE1-YBR241C*	4.90E-06	Y
78	*LCB2-TRA1*	4.90E-06	Y
79	*POL2-RAD27*	4.95E-06	Y
80	*ATG18-POP4*	4.97E-06	Y
81	*BRR2-LSC2*	4.98E-06	Y
82	*ARP3-PEP8*	5.00E-06	Y
83	*GCD6-PEP7*	5.13E-06	Y
84	*CTF18-FAL1*	5.15E-06	Y
85	*COQ3-YDC1*	5.26E-06	Y
86	*BUD32-FET5*	5.38E-06	N
87	*KAR3-MLH3*	5.41E-06	Y
88	*AGE2-SSU72*	5.43E-06	Y
89	*EFT1,EFT2-RPS2*	5.44E-06	Y
90	*ARG4-GLR1*	5.66E-06	Y
91	*PTC3-YEF1*	5.69E-06	N
92	*GLE1-IML1*	5.85E-06	Y
93	*COQ3-YPK9*	5.86E-06	Y
94	*SLM3-YIP5*	5.87E-06	Y
95	*ATG26-KAR2*	5.93E-06	Y
96	*DUS3-MDJ1*	6.01E-06	N

97	*CDC33-UNG1*	6.14E-06	N
98	*ENP2-PUP2*	6.28E-06	Y
99	*ERO1-LSM12*	6.37E-06	Y
100	*MES1-SPF1*	6.37E-06	Y
Y-M_f			
1	*CUP5-TUB2*	1.14E-07	Y
2	*MSH4-MSH5*	1.56E-07	N
3	*CDC28-CUP5*	2.49E-07	N
4	*PDA1-SRP102*	2.69E-07	Y
5	*IRC24-SLM3*	3.89E-07	Y
6	*CAB1-KCS1*	6.23E-07	Y
7	*RPA135-VPS15*	6.60E-07	N
8	*STE4-YGR127W*	6.69E-07	Y
9	*ASC1-RPP2B*	7.93E-07	N
10	*RRI1-TID3*	1.08E-06	N
11	*CTR9-VPS74*	1.11E-06	Y
12	*BOI2-HRD1*	1.13E-06	Y
13	*PEX2-TPC1*	1.15E-06	Y
14	*UGP1-YAT1*	1.32E-06	Y
15	*IPP1-YCR090C*	1.36E-06	Y
16	*KAP104-RPC37*	1.39E-06	Y
17	*HEM2-MIA40*	1.39E-06	Y
18	*RPS10A-SFA1*	1.40E-06	Y
19	*NAT1-NBP35*	1.44E-06	Y
20	*DOC1-MSH4*	1.49E-06	N
21	*RER2-RPL25*	1.66E-06	Y
22	*CAT5-UTP13*	1.69E-06	Y
23	*BRF1-LSC2*	1.75E-06	N
24	*HRD1-RPN1*	1.78E-06	N
25	*FMP37-TAD1*	1.95E-06	Y
26	*GOR1-YPL225W*	2.03E-06	N
27	*MLP1-YFR018C*	2.08E-06	N
28	*TFG2-YHR020W*	2.14E-06	Y
29	*MAS1-RBD2*	2.17E-06	Y
30	*ARP2-LPD1*	2.21E-06	Y
31	*PRE1-SMC6*	2.23E-06	N
32	*ALG5-SGS1*	2.26E-06	Y
33	*NAT1-ROT2*	2.27E-06	Y
34	*DCN1-PTH2*	2.33E-06	Y
35	*ARF2-SYP1*	2.39E-06	Y
36	*PTC3-TSR2*	2.44E-06	Y
37	*NPA3-TPT1*	2.46E-06	Y
38	*NSE1-ZTA1*	2.50E-06	Y
39	*RPT4-YEF1*	2.56E-06	Y
40	*ATG12-EXO70*	2.60E-06	Y
41	*FMP32-LSM12*	2.63E-06	N
42	*SED5-YDL114W*	2.66E-06	Y
43	*POR1-YKR070W*	2.76E-06	Y
44	*CKB2-NSP1*	2.77E-06	Y
45	*MSS4-TFP1*	2.82E-06	Y

46	*SMC5-TCB3*	2.83E-06	Y
47	*IFM1-MTO1*	2.89E-06	N
48	*CHK1-ECM29*	3.06E-06	Y
49	*RRI1-TOP2*	3.08E-06	Y
50	*RAD26-ROM2*	3.09E-06	Y
51	*ATG18-RRI1*	3.11E-06	Y
52	*CRG1-DIP2*	3.18E-06	Y
53	*LSC2-RPL43A,RPL43B*	3.26E-06	Y
54	*COX17-RPB2*	3.56E-06	Y
55	*MCM2-RRN3*	3.58E-06	N
56	*MPE1-PRP22*	3.70E-06	Y
57	*APT1-ATP16*	3.71E-06	N
58	*CDC42-MTQ1*	3.81E-06	Y
59	*SPP382-VID24,YGR066C*	3.95E-06	Y
60	*GLY1-RRP6*	3.97E-06	Y
61	*SLM3-YVH1*	4.06E-06	Y
62	*DED1-RTS2*	4.17E-06	N
63	*ALG2-NBP35*	4.22E-06	Y
64	*DUS3-MDJ1*	4.27E-06	N
65	*GOS1-RIM11*	4.33E-06	N
66	*FOX2-HRD1*	4.34E-06	N
67	*RPS25A-ZUO1*	4.49E-06	Y
68	*MIG1-SEC6*	4.53E-06	Y
69	*PKH2-TAF9*	4.56E-06	Y
70	*VTI1-YAK1*	4.56E-06	Y
71	*PUT1-YOR289W*	4.59E-06	N
72	*GET3-SRP102*	4.68E-06	N
73	*NIF3-RIM15*	4.69E-06	N
74	*PHO87,PHO90,PHO91-RRP3*	4.77E-06	Y
75	*PIS1-RRP40*	4.93E-06	Y
76	*IMP2-JAC1*	4.94E-06	Y
77	*DAL81-YPL191C*	5.09E-06	Y
78	*SIT4-VPS21*	5.18E-06	Y
79	*ERS1-RAD51*	5.30E-06	Y
80	*KAR3-RPN7*	5.48E-06	N
81	*SSM4-TAF6*	5.49E-06	Y
82	*ATG8-RSP5*	5.51E-06	Y
83	*RPO41-SAL1*	5.68E-06	Y
84	*NFU1-RBG1*	5.72E-06	N
85	*BET4-NMA111*	5.79E-06	Y
86	*PMS1-YDR374C*	5.82E-06	Y
87	*GRR1-LSC2*	5.83E-06	Y
88	*AIM17-SCP1*	5.89E-06	Y
89	*DYN2-YSH1*	5.98E-06	Y
90	*DBP8-POL4*	6.05E-06	Y
91	*EHD3-KGD1*	6.10E-06	Y
92	*CDC33-UNG1*	6.13E-06	N
93	*DRS2-TFC4*	6.13E-06	Y
94	*RKM4-RPS19A*	6.15E-06	Y
95	*MSY1-SSC1*	6.21E-06	Y

96	*CDC3-HRD1*	6.23E-06	Y
97	*CDC39-CEG1*	6.29E-06	Y
98	*COX10-SLP1*	6.36E-06	Y
99	*DIA4-SRP72*	6.46E-06	N
100	*VPS16-VPS29*	6.75E-06	Y
Y-H_f			
1	*RPN7-SDH2*	1.17E-08	N
2	*HOP1-SPO11*	8.54E-08	Y
3	*TLG1-TYW3*	1.72E-07	N
4	*BUD32-FET5*	2.21E-07	N
5	*YBR241C-YNL155W*	2.96E-07	N
6	*POB3-SCP160*	4.79E-07	N
7	*DDP1-SPB1*	5.45E-07	N
8	*BZZ1-PRP16*	7.98E-07	N
9	*GIM4-SHP1*	8.02E-07	Y
10	*DIP2-QRI1*	8.14E-07	Y
11	*RPT5-SFH1*	8.63E-07	Y
12	*SCC2-SPO22*	8.93E-07	Y
13	*IDH2-ORC5*	9.81E-07	Y
14	*IDH2-TAF5*	9.97E-07	Y
15	*GET3-SRP102*	1.12E-06	N
16	*CCT3-RPT6*	1.15E-06	Y
17	*CUL3-PCM1*	1.24E-06	N
18	*NIF3-RIM15*	1.28E-06	N
19	*GLE1-QRI1*	1.30E-06	Y
20	*AKL1,ARK1-RNA14*	1.34E-06	Y
21	*DOC1-SCC2*	1.39E-06	Y
22	*CDC28-CUP5*	1.41E-06	N
23	*CCT3-RPN12*	1.41E-06	Y
24	*SRO9-YNL305C*	1.62E-06	Y
25	*KIC1-RAM2*	1.71E-06	Y
26	*PYC1-SSU72*	1.74E-06	N
27	*APL4-TAF5*	1.75E-06	Y
28	*DED1-RTS2*	1.78E-06	N
29	*HCS1-RGA2*	1.80E-06	Y
30	*CUL3-MET30*	1.86E-06	Y
31	*DUS3-MDJ1*	1.90E-06	N
32	*CYS3-SCY1*	1.92E-06	Y
33	*HOP1-SPO22*	2.22E-06	Y
34	*RPO41-RPT4*	2.24E-06	Y
35	*TIM23-YUH1*	2.27E-06	Y
36	*ETP1-YAT1*	2.34E-06	Y
37	*SCC2-SPO11*	2.38E-06	Y
38	*MSH5-SCC2*	2.46E-06	N
39	*PDA1-SCC2*	2.50E-06	Y
40	*IDH2-UTP4*	2.70E-06	N
41	*PTC7-UBA2*	2.74E-06	Y
42	*UFD2-VPS24*	2.80E-06	Y
43	*LRG1-ROM2*	2.96E-06	Y
44	*ARL3-BCP1*	3.09E-06	Y

45	*CSE1-SUI2*	3.29E-06	Y
46	*BRF1-LSC2*	3.34E-06	N
47	*IRA1-TCP1*	3.37E-06	Y
48	*HAH1-MRPS9*	3.44E-06	Y
49	*MCM5-RNH201*	3.80E-06	Y
50	*CGI121-NIF3*	3.84E-06	N
51	*PEX14-RAD50*	3.95E-06	Y
52	*CCT2-UTP4*	4.00E-06	Y
53	*CCT7-RAD27*	4.03E-06	Y
54	*CSE1-DIP2*	4.18E-06	Y
55	*ADA2-YAF9*	4.20E-06	Y
56	*PUT1-YOR289W*	4.27E-06	N
57	*PEX12-YKR017C*	4.28E-06	Y
58	*CUL3-YLR118C*	4.29E-06	Y
59	*ALG13-CYC3*	4.32E-06	Y
60	*ATG7-TFA2*	4.34E-06	Y
61	*CDC33-UNG1*	4.46E-06	N
62	*PNS1-VRG4*	4.47E-06	Y
63	*RSR1-YLR118C*	4.50E-06	Y
64	*TPC1-TRM5*	4.69E-06	Y
65	*MIP1-TFA2*	4.76E-06	Y
66	*DMC1-HOP1*	4.94E-06	N
67	*PEX13-SPO22*	4.96E-06	Y
68	*FCP1-RPC53*	5.21E-06	Y
69	*COX10-YKU70*	5.26E-06	Y
70	*MAK10-MAK5*	5.56E-06	N
71	*ATG7-MIP1*	5.58E-06	Y
72	*FAT1-GFA1*	5.70E-06	Y
73	*CYT1-RAD27*	5.76E-06	Y
74	*SOD1-STP22*	5.85E-06	Y
75	*AGX1-YMR074C*	5.89E-06	N
76	*CBF5-TSR2*	5.93E-06	Y
77	*DOC1-PEX13*	6.00E-06	Y
78	*BUB1-TCP1*	6.13E-06	Y
79	*RVB2-YHP1*	6.18E-06	Y
80	*APL4-MCM6*	6.29E-06	Y
81	*ALT2-NIC96*	6.34E-06	Y
82	*GRX2-LRG1*	6.37E-06	Y
83	*MCM2-RRN3*	6.52E-06	N
84	*RRI1-TID3*	6.58E-06	N
85	*ETR1-MRPS9*	6.65E-06	Y
86	*DIP2-DMC1*	6.73E-06	Y
87	*QRI1-RPN2*	6.73E-06	N
88	*ETP1-GCD10*	6.74E-06	Y
89	*QRI7-SUA5*	6.76E-06	Y
90	*ERG26-MAG2*	6.99E-06	Y
91	*DAP2-ROM2*	7.23E-06	Y
92	*SPO11-SPO22*	7.27E-06	Y
93	*BOR1-PNS1*	7.32E-06	Y
94	*FCP1-SET1*	7.39E-06	Y

95	*CCT2-CCT4*	7.51E-06	Y
96	*CSE1-PEX13*	7.54E-06	Y
97	*ESS1-RIX7*	7.68E-06	Y
98	*MCM6-QRI1*	7.70E-06	Y
99	*RAD27-RPT6*	7.82E-06	N
100	*IZH2-RFC4*	7.88E-06	Y
$Y\text{-}M_m\text{-}M_f\text{-}H_f$			
1	*NFU1-RBG1*	8.08E-10	N
2	*PUF6-RVB1*	6.15E-09	N
3	*RPN7-SDH2*	8.58E-09	N
4	*KAR3-RPN7*	1.38E-08	N
5	*CYC3-SNF4*	1.72E-08	Y
6	*HRD1-RPN1*	3.25E-08	N
7	*FOL2-RRP45*	4.24E-08	Y
8	*COG6-HOP1*	5.87E-08	N
9	*CGI121-NIF3*	6.12E-08	N
10	*POB3-SCP160*	6.58E-08	N
11	*DIA4-SRP72*	7.90E-08	N
12	*DED1-RTS2*	8.42E-08	N
13	*COX17-PAM16*	8.55E-08	Y
14	*NAT1-SPT15*	9.11E-08	Y
15	*LAP2-YBR241C*	9.95E-08	Y
16	*ARG5,6-ATP12*	9.96E-08	Y
17	*GOS1-RIM11*	9.97E-08	N
18	*PRO3-UTP14*	1.04E-07	Y
19	*UTP4-YAF9*	1.14E-07	Y
20	*APT1-ATP16*	1.17E-07	N
21	*RPL32-RPL37A*	1.19E-07	N
22	*MAK10-MAK5*	1.24E-07	N
23	*CDC28-CUP5*	1.42E-07	N
24	*MSH5-SCC2*	1.60E-07	N
25	*MCM7-SCP160*	1.60E-07	Y
26	*UBC7-YUH1*	1.67E-07	Y
27	*MCM4-SMC2*	1.73E-07	Y
28	*BUD32-FET5*	1.80E-07	N
29	*PNG1-SPT15*	1.82E-07	N
30	*DOC1-RPN2*	1.84E-07	Y
31	*ASC1-RPP2B*	1.93E-07	N
32	*ALF1-IMP4*	1.94E-07	Y
33	*CUL3-PCM1*	2.00E-07	N
34	*DMC1-HOP1*	2.03E-07	N
35	*MDJ1-NOP4*	2.05E-07	Y
36	*ADA2-ARP3*	2.06E-07	Y
37	*RPL19A,RPL19B-RPS8A,RPS8B*	2.10E-07	Y
38	*GPI8-VPS35*	2.14E-07	Y
39	*EBP2-NOT3*	2.17E-07	Y
40	*CAX4-SKY1*	2.19E-07	Y
41	*CUS1-NSE4*	2.32E-07	Y
42	*POB3-RPF1*	2.33E-07	Y

43	*UBC7-VPS35*	2.36E-07	Y
44	*GLO2-RPH1*	2.67E-07	Y
45	*FMP43-YMR074C*	2.68E-07	Y
46	*IDH2-IPP1*	2.69E-07	Y
47	*BUB1-MLH1*	2.72E-07	Y
48	*TSA1-YNL155W*	2.73E-07	Y
49	*AOS1-SIS1*	2.81E-07	Y
50	*APL4-IDH2*	2.86E-07	Y
51	*DDP1-SPB1*	2.95E-07	N
52	*NAM8-STE24*	2.96E-07	N
53	*RPL2A,RPL2B-RPP2B*	2.96E-07	Y
54	*CYC3-ZRC1*	3.00E-07	Y
55	*CDC50-RRP1*	3.08E-07	N
56	*FOX2-HRD1*	3.13E-07	N
57	*SOD2-SUA5*	3.20E-07	Y
58	*CUL3-SUI2*	3.31E-07	Y
59	*FMP32-LSM12*	3.39E-07	N
60	*CCT5-YKE2*	3.71E-07	N
61	*COG3-CYB5*	3.72E-07	N
62	*IFM1-MTO1*	3.76E-07	N
63	*GEM1-VPS45*	3.77E-07	Y
64	*GET3-SRP102*	3.78E-07	N
65	*BNA6-FMP41*	3.82E-07	Y
66	*NIF3-RIM15*	3.96E-07	N
67	*RPA135-VPS15*	4.02E-07	N
68	*RAD27-RPT6*	4.09E-07	N
69	*CCT4-PAM16*	4.26E-07	Y
70	*YIP3-YUH1*	4.28E-07	Y
71	*ATP4-SSL1*	4.46E-07	Y
72	*DIP2-GLE1*	4.75E-07	Y
73	*NSE4-SUB2*	4.75E-07	Y
74	*BUB1-QRI1*	4.83E-07	Y
75	*HMT1-NHP2*	4.85E-07	Y
76	*DOA1-TOM1*	4.86E-07	Y
77	*LSB6-QRI1*	4.92E-07	Y
78	*MAF1-TOP2*	4.93E-07	Y
79	*GID7-LPD1*	5.01E-07	Y
80	*OCT1-TFA1*	5.10E-07	Y
81	*PTC3-YEF1*	5.28E-07	N
82	*POR1-TIM23*	5.29E-07	Y
83	*QRI1-RPN2*	5.44E-07	N
84	*TLG1-TYW3*	5.53E-07	N
85	*RPL18A,RPL18B-RPL2A,RPL2B*	5.54E-07	Y
86	*IDH2-UTP4*	5.57E-07	N
87	*DPP1-KAP114*	5.58E-07	N
88	*YBR241C-YNL155W*	5.62E-07	N
89	*LSM1-RVB1*	5.63E-07	Y
90	*BZZ1-PRP16*	5.76E-07	N
91	*ORC5-VMA8*	5.99E-07	Y

92	*CDC6-DIP2*	6.11E-07	Y
93	*RPT6-SVP26*	6.23E-07	Y
94	*AAH1-YNL024C*	6.67E-07	Y
95	*ATM1-TMA46*	6.82E-07	Y
96	*RRS1-RSE1*	7.00E-07	Y
97	*CCT3-CCT4*	7.01E-07	Y
98	*CDC50-TOP2*	7.03E-07	Y
99	*MTC5-TLG1*	7.06E-07	N
100	*TOM1-YET3*	7.10E-07	Y

* Top metagene pairs are the pairs with the most significant P-values for co-expression across species.

Table S5 – Method comparison by calculating the enrichment of meiosis term GO:0007126 in yeast genes

	Order statistics		Hierarchical clustering	
	Gene number*	Hypergeometric P-value	Gene number#	Hypergeometric P-value
$Y\text{-}M_m$	174	0.001	142	0.03
$Y\text{-}M_f$	191	0.019	88	0.002
$Y\text{-}H_f$	154	0.009	179	0.001
$Y\text{-}M_m\text{-}M_f\text{-}H_f$	171	0.033	91	0.005

* Top-100 metagene pairs with the most significant order statistics P-values were used for analysis. Gene number is the number of yeast genes in the top-100 metagene pairs.

# The cluster with the most significant hypergeometric P-value of meiosis GO term is listed here. Gene number is the number of yeast genes in that cluster.
